# Visual Neurons in the Superior Colliculus Innervated by Islet2^+^ or Islet2^−^ Retinal Ganglion Cells Display Distinct Tuning Properties

**DOI:** 10.3389/fncir.2017.00073

**Published:** 2017-10-10

**Authors:** Rachel B. Kay, Jason W. Triplett

**Affiliations:** ^1^Center for Neuroscience Research, Children’s National Medical Center, Washington, DC, United States; ^2^Department of Pediatrics, The George Washington University School of Medicine and Health Science, Washington, DC, United States

**Keywords:** visual system, receptive field, mouse, direction-selective, orientation-selective

## Abstract

Throughout the visual system, different subtypes of neurons are tuned to distinct aspects of the visual scene, establishing parallel circuits. Defining the mechanisms by which such tuning arises has been a long-standing challenge for neuroscience. To investigate this, we have focused on the retina’s projection to the superior colliculus (SC), where multiple visual neuron subtypes have been described. The SC receives inputs from a variety of retinal ganglion cell (RGC) subtypes; however, which RGCs drive the tuning of different SC neurons remains unclear. Here, we pursued a genetic approach that allowed us to determine the tuning properties of neurons innervated by molecularly defined subpopulations of RGCs. In homozygous *Islet2-EphA3* knock-in (Isl2^EA3/EA3^) mice, Isl2^+^ and Isl2^−^ RGCs project to non-overlapping sub-regions of the SC. Based on molecular and anatomic data, we show that significantly more Isl2^−^ RGCs are direction-selective (DS) in comparison with Isl2^+^ RGCs. Targeted recordings of visual responses from each SC sub-region in Isl2^EA3/EA3^ mice revealed that Isl2^−^ RGC-innervated neurons were significantly more DS than those innervated by Isl2^+^ RGCs. Axis-selective (AS) neurons were found in both sub-regions, though AS neurons innervated by Isl2^+^ RGCs were more tightly tuned. Despite this segregation, DS and AS neurons innervated by Isl2^+^ or Isl2^−^ RGCs did not differ in their spatial summation or spatial frequency (SF) tuning. Further, we did not observe alterations in receptive field (RF) size or structure of SC neurons innervated by Isl2^+^ or Isl2^−^ RGCs. Together, these data show that innervation by Isl2^+^ and Isl2^−^ RGCs results in distinct tuning in the SC and set the stage for future studies investigating the mechanisms by which these circuits are built.

## Introduction

Perception of the visual world is a complex neural computation mediated by different subtypes of neurons that monitor distinct aspects of the visual scene. For example, retinal ganglion cells (RGCs) relay all visual information to the brain and can be subdivided into a diverse array of subtypes tuned to specific visual features (Sanes and Masland, 2015). As RGCs project to central nuclei, precise synaptic connections must be established to faithfully relay these distinct bits of visual information and establish novel tuning properties. While the subtypes of RGCs (Baden et al., 2016) and subtypes of neurons in retinorecipient regions (Wang et al., 2010; Marshel et al., 2012; Piscopo et al., 2013; Gale and Murphy, 2014; Ito et al., 2017) have been described, the RGCs are responsible for driving different tuning properties in these areas remains unclear.

A primary image-forming target of RGC innervation is the superior colliculus (SC), a midbrain nucleus that regulates goal-directed, saccadic eye movements (Wurtz and Albano, 1980; May, 2006; Krauzlis et al., 2013). Multiple subtypes of RGCs project to the SC (Vaney et al., 1981; Gauvain and Murphy, 2015; Ito et al., 2017) and neurons in the SC exhibit similar tuning to those RGCs, as well as novel tuning properties. For example, while RGCs have center-surround organized receptive fields (RFs; Kuffler, 1953), most neurons in the SC have overlapping On- and Off- domains (Drager and Hubel, 1975; Wang et al., 2010). Furthermore, direction-selective (DS) and axis-selective (AS) neurons in the SC exhibit preferences to all directions, while directionally tuned RGCs (DSGCs) only prefer cardinal directions (Wang et al., 2010; Inayat et al., 2015; Nath and Schwartz, 2016).

How the tuning of neurons in retinorecipient regions arises has been an intense area of investigation recently. In the dorsal lateral geniculate nucleus (dLGN), the enrichment of DS neurons in regions densely innervated by genetically labeled DSGCs suggests tuning may be inherited from the retina (Huberman et al., 2009; Piscopo et al., 2013). Direct functional evidence has demonstrated a similar mechanism to establish direction-selectivity in the SC (Shi et al., 2017). In contrast, the prevalence of AS neurons in DSGC-innervated regions of the dLGN (Marshel et al., 2012) and diversity of RGC subtypes connected to individual dLGN neurons (Rompani et al., 2017) suggest that more complex wiring strategies may underlie specific tuning. These studies have advanced our understanding, but the molecular identity of RGCs driving different tuning remains unclear.

Previously, we demonstrated that RGCs expressing the transcription factor Islet2 (Isl2) are comprised of related subsets of RGCs that share morphological characteristics (Triplett et al., 2014). Specifically, the dendrites of Isl2^+^ RGCs are predominantly mono-stratified in a specific sublayer of the inner plexiform layer (IPL) of the retina, and none display the well-established bistratified morphology characteristic of On-Off DSGCs. Furthermore, a substantial portion of Isl2^+^ RGCs express the phosphoprotein SMI-32, a marker of alpha-type RGCs (Coombs et al., 2006). In contrast, many Isl2^−^ RGCs are bistratified, none express SMI-32, and at least one subtype of genetically labeled On-Off DSGCs is Isl2^−^. These data suggest that Isl2^+^ and Isl2^−^ RGCs relay distinct functional information to the SC, potentially relating to object recognition and motion discrimination, respectively. However, it remains unclear how Isl2^+^ and Isl2^−^ RGCs influence the tuning of their downstream partners.

Here, we utilized the homozygous *Islet2-EphA3* knock-in mice (Isl2^EA3/EA3^), in which Isl2^+^ and Isl2^−^ RGCs project to non-overlapping sub-regions of the SC (Brown et al., 2000; Triplett et al., 2014), to determine how Isl2^+^ and Isl2^−^ RGCs influence the tuning properties of SC neurons. We show that distinct functional information is relayed to each sub-region, because the terminals of genetically labeled subclasses of DSGCs are restricted to the Isl2^−^ RGC-innervated sub-region, and we found that significantly more Isl2^−^ RGCs express a marker of On-Off DSGCs. These data were consistent with our previous findings and suggest that multiple DSGCs are Isl2^−^, raising the possibility that SC neurons innervated by these RGCs are more likely to be DS. Indeed, we found significantly more DS neurons in the Isl2^−^ RGC-innervated sub-region and a significant decrease in direction-selectivity in the Isl2^+^ RGC-innervated sub-region. Conversely, we identified AS neurons in both subregions, though those innervated by Isl2^+^ RGCs were more highly selective than those innervated by Isl2^−^ RGCs. Despite this segregation, we found no significant differences in the spatial summation and spatial frequency (SF) tuning properties of DS and AS neurons in each sub-region. We further observed no alterations in RF size nor changes in overlap of On and Off subfields. Taken together, these data demonstrate that Isl2^+^ and Isl2^−^ RGCs relay distinct visual information to the SC and influence different tuning properties in the SC. These findings set the stage for future studies of the molecular mechanisms underlying the development of subtype-specific connectivity.

## Materials and Methods

### Mice

*Islet2-EphA3* knock-in and thyrotropin-releasing hormone receptor (TRHR)-GFP mice were generated and genotyped as previously described (Brown et al., 2000; Rivlin-Etzion et al., 2011). Mice were maintained on a mixed 129S1/C57BL6 background. Mice were housed in a temperature and humidity controlled room under standard 12/12 h light-dark cycle. After weaning, mice were housed in groups of 1–5 with same-sex siblings. This study was carried out in accordance with the recommendations of the Guide for the Care and Use of Laboratory Animals by the National Research Council. The protocol was approved by the Institutional Animal Care and Use Committee at Children’s National Medical Center.

### Immunohistochemistry

Postnatal mice were sacrificed and intracardially perfused with ice-cold phosphate buffered saline (in mM: 136.9 NaCl, 2.7 KCl, 10.1 Na_2_HPO_4_, 1.4 K_2_PO_4_; PBS) followed by ice-cold paraformaldehyde (PFA; pH 7.4, 4% in PBS). Eyes and brains were dissected out and fixed in 4% PFA for either 30 min at room temperature or overnight at 4°C, respectively. Tissue was washed briefly in PBS and sunk in 30% sucrose overnight at 4°C. The following day, tissue was embedded in Tissue-Tek OCT Compound (Sakura Finetek, Torrance, CA, USA) on dry ice and stored at −80°C until sectioned. For retinas, thin sections were cut at 16–20 μm on a CM1520 Cryostat (Leica Microsystems, Buffalo Grove, IL, USA) maintained at −20 to −25°C and collected on histology-grade glass slides. Slides were allowed to dry overnight at room temperature and immediately used for immunostaining or frozen at −80°C until used. For brains, 100 μm sections were cut on a sliding microtome (Leica Microsystems) and free-floating sections were processed for immunostaining immediately. The following antibodies were diluted as indicated in blocking buffer and incubated overnight at 4°C: GFP rabbit polyclonal, 1:1000 (Invitrogen, Carlsbad, CA, USA); cocaine and amphetamine related transcript (CART) rabbit polyclonal (Phoenix Pharmaceuticals, Burlingame, CA, USA). Imaging was performed with an Olympus BX61 epifluorescent microscope (Olympus, Center Valley, PA, USA) equipped with Olympus DP71 digital camera (Olympus).

### Axon Tracing

#### Intraocular Injections to Anterogradely Trace Retinofugal Axons

To label all retinofugal axons, adult mice were anesthetized by subcutaneous injection of ketamine/xylazine solution (100/10 mg/kg). Approximately 1 μL of fluorescently-conjugated cholera toxin subunit B (CTB-555, Invitrogen, Carlsbad, CA, USA; 2 mg/mL) was injected into the SC using a pulled glass pipet and Picospritzer III (Parker-Hannafin) set at high pressure (~30 psi) and short pulse duration (~15 ms). After 2–3 days, mice were sacrificed and intracardially perfused with PBS and PFA. Brains were dissected out and fixed overnight in 4% PFA at 4°C. The following day, brains were briefly washed with PBS and cryopreserved in 30% sucrose at 4°C overnight. Sagittal sections were cut at 100 μm with an HM430 sliding microtome (Thermo-Fisher, Waltham, MA, USA) and collected in PBS. Immunostaining for GFP was performed as described above for whole mount retinas.

#### Intracolllicular Injections to Retrogradely Label RGC Cell Bodies

To retrogradely label RGCs, adult mice were anesthetized with ketamine/xylazine (100/10 mg/kg i.p.) and placed in a stereotaxic frame. A small incision was made in the scalp to expose the skull and a focal craniotomy was performed with a microdrill. A Hamilton syringe with 33 gauge needle attached to a Quintessential Stereotaxic Injector (Stoelting, Wood Dale, IL, USA) was lowered into the SC at an angle of 30° to the vertical and to a depth of 1–1.5 mm. Approximately 1 μL of fluorescently-conjugated cholera toxin subunit B (CTB-488, Invitrogen; 10 mg/mL in PBS) was injected at a rate of 0.5 mL/min. The scalp was sutured and mice were allowed to recover in their home cage on a warming plate. After 2 days, mice were sacrificed and intracardially perfused with PBS and PFA. Eyes were dissected out and prepared for imaging as described above.

### *In Vivo* Electrophysiology

Adult mice (>P40) of both sexes were anesthetized with isoflurane. The animal’s temperature was monitored and maintained at 37°C through a feedback-controlled heating pad. Silicone oil was applied on the eyes to prevent drying. A craniotomy was performed on the right hemisphere ~1 mm lateral from the midline suture, and between 1.5 mm and −0.25 mm anterior from the lambda suture. Agarose (1% in PBS) was placed on the exposed brain tissue. A 16-channel silicone probe (Neuronexus) coated in 1,1′-dioctadecyl-3,3,3′,3′-tetramethylindocarbocyanine (DiI; Invitrogen) was lowered between 0.8 mm and 1.5 mm into the SC at a 35° angle. Electrical signals were filtered between 0.7 kHz and 7 kHz, sampled at 25 kHz, and acquired using a System 3 workstation (Tucker-Davis Technologies). At the conclusion of the experiment, animals were perfused with PBS followed by 4% PFA and brains were sagittally sectioned to confirm location of the electrode in either the anterior or posterior SC.

### Visual Stimuli

Visual stimuli were generated with customized Matlab programs (Niell and Stryker, 2008). The monitor (52 cm × 29.5 cm, 60 Hz refresh rate) was placed 22 cm from the animal in front of the eye contralateral to the recording penetration. To calculate RF size, 5° × 5° white squares were flashed at different locations on a 27 × 17 grid with a gray background, thus the resolution of our analysis is 5° of visual space and maximum error is 25 degree^2^. The squares were on for 500 ms and off for 500 ms at pseudorandom locations for five trials at each location. Response field sizes were calculated by fitting a two-dimensional Gaussian distribution as described in Wang et al. (2010). The response ratio was calculated with the peak On and Off responses (*R*_on, max_ − *R*_off, max_)/(*R*_on, max_ + *R*_off, max_). The overlap index measures the degree of overlap between the On and Off subfields while taking into account the distance between their centers. It is calculated using the equation: OI = (*W*_1_ + *W*_2_ − *c*)/(*W*_1_ + *W*_2_ + *c*), where *W*_1_ and *W*_2_ are the half-widths of the subfields measured along the line joining the subfield centers and *c* is the distance between the On and Off subfield centers (Wang et al., 2010).

To calculate directional and axial tuning, drifting gratings of 100% contrast at 12 different directions (30° spacing) and six different SFs between 0.01 and 0.32 cycles per degree (cpd; six logarithmic steps). A temporal frequency of 2 Hz was consistent for all the gratings. Each stimulus of given direction and SF (or a blank condition) was presented for 1.5 s in a pseudorandom order for five trials. The interval between stimuli was 0.5 s. The preferred direction (θ_pref_) was determined as the one that evoked maximum response. The modulation was described using two parameters: (1) direction selectivity index (DSI) = (*R*_pref_ − *R*_opp_)/(*R*_pref_ + *R*_opp_), where R_pref_ was the response at θ_pref_ and *R*_opp_ at θ_pref_+π; and (2) orientation selectivity index (OSI) = (*R*_pref_ − mean(*R*_orth_))/(*R*_pref_ + mean(*R*_orth_)). The tuning curves were fitted with a sum of two Gaussians centered at θ_pref_ and θ_pref_+π using the nlinfit function in Matlab, and the tuning width was calculated as the half-width at half-maximum of the fitted curve above the baseline (Niell and Stryker, 2008). A cell was considered DS with a DSI greater than 0.33 and OSI greater than 0.33. A cell was considered AS with an OSI greater than 0.33 and a DSI less than 0.33.

Linearity of response to drifting gratings was calculated at the preferred direction and SF by first binning the response at 100 ms intervals (minus the spontaneous rate) and then applying a discrete Fourier transform to compute F_1_/F_0_, the ratio of the first harmonic (response at the drift frequency of 2 Hz) to the mean response (Niell and Stryker, 2008). A F_1_/F_0_ ratio greater than 1 was considered linear (Skottun et al., 1991).

### Statistical Analysis

Statistical analyses and graph plotting were done with Prism (GraphPad Software) and Matlab (MathWorks). Statistical tests used for each data set were determined based on the distribution of the data and number of groups, and are indicated in the text and figure legends. All data are reported as mean ± SEM.

## Results

**Figure 1:**
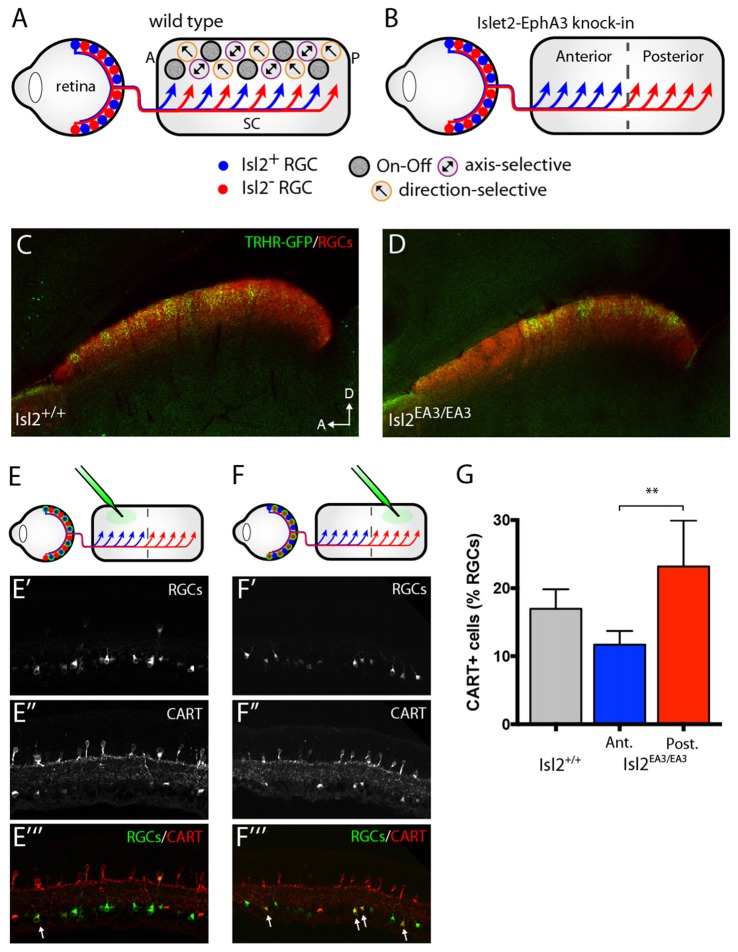
Segregation of Isl2^+^ and Isl2^−^ retinal ganglion cell (RGC) inputs in the superior colliculus (SC) of Isl2^EA3/EA3^ mice. **(A,B)** Schematics depicting retinocollicular projections and SC receptive field (RF) properties in the SC of Isl2^+/+^ **(A)** and Isl2^EA3/EA3^ **(B)** mice. In the Isl2^+/+^ SC, Isl2^+^ and Isl2^−^ cells project throughout the anterior-posterior (A-P) axis of the SC and On-Off, direction-selective (DS) and axis-selective (AS) cells are found throughout the SC. In *Islet2-EphA3* knock-in (Isl2^EA3/EA3^) mice Isl2^+^ RGCs terminate predominantly in the anterior portion of the SC and Isl2^−^ RGCs terminate predominantly in the posterior. The RF properties of neurons innervated by these populations are unknown. **(C,D)** Parasagittal sections through the SC of adult (P30) Isl2^+/+^ **(C)** and Isl2^EA3/EA3^ mice **(D)** in which all RGC terminations are labeled by CTB-555 (red) and thyrotropin-releasing hormone receptor (TRHR)-GFP RGC terminations are labeled by immunofluorescence of GFP (green). **(E,F)** *top* Schematics depicting strategy to retrogradely label RGCs projecting to the anterior **(E)** or posterior **(F)** SC of Isl2^EA3/EA3^ mice. *bottom* Cross-sections of retinas in which CTB-488 (green) was injected into the anterior or posterior SC to label RGCs **(E′,F′)** and immunofluorescently labeled for cocaine and amphetamine related transcript (CART) **(E″,F″)**, and merged image to reveal co-localization **(E″′,F″′)**. **(G)** Quantification of the percent of retrogradely labeled RGCs co-stained positively for the putative On-Off DSGC marker CART. **p < 0.01, One-Way ANOVA and Tukey’s *post hoc* analysis.

To explore the wiring strategies underlying visual tuning, we utilized a unique transgenic mouse line in which the terminals of Isl2^+^ and Isl2^−^ RGCs are segregated into non-overlapping domains of the SC. In Isl2^EA3/EA3^ mice, the receptor tyrosine kinase EphA3 is exogenously overexpressed from the Isl2 locus (Brown et al., 2000). Isl2 is a transcription factor expressed in ~40% of RGCs (Pak et al., 2004); thus, in Isl2^EA3/EA3^ mice, two populations of RGCs can be distinguished: Isl2^+^ RGCs that express exogenously high levels of EphA and Isl2^−^ RGCs that express endogenous levels. Since the relative level of EphA expression regulates termination location along the anterior-posterior (A-P) axis of the SC (Triplett, 2014), in Isl2^EA3/EA3^ mice, the anterior half of the SC is innervated exclusively by Isl2^+^ RGCs, while the posterior half is innervated exclusively by Isl2^−^ RGCs (Figures 1A,B). Indeed, in Isl2^EA3/EA3^ mice, two distinct anatomical and functional topographic maps can be identified in the SC (Brown et al., 2000; Triplett et al., 2009). Importantly, our previous studies suggest that over-expression of EphA3 does not alter the proportions, dendritic lamination properties, nor axonal targeting of genetically-labeled RGCs, suggesting that functionality of RGCs is minimally altered in Isl2^EA3/EA3^ mice (Triplett et al., 2014). Intriguingly, we also previously found that though each functional map occupies the same proportion of the SC in Isl2^EA3/EA3^ mice, the population response properties to a drifting bar stimulus were significantly different (Triplett et al., 2009). These data raise the possibility that SC neurons innervated by Isl2^+^ and Isl2^−^ RGCs have differential tuning properties, a hypothesis we tested here.

### Segregation of DSGC Inputs into the Posterior SC of Isl2^EA3/EA3^ Mice

Previously, we showed that the terminals of genetically labeled dopamine receptor 4 (DRD4)-GFP DSGCs preferring posterior direction were restricted to the posterior domain of the SC in Isl2^EA3/EA3^ mice (Triplett et al., 2014). To determine if other DSGCs may also project predominantly to this domain, we crossed the TRHR-GFP line, which marks a distinct subset of DSGCs (Rivlin-Etzion et al., 2011), into the Isl2^EA3/EA3^ background. Consistent with previous results, we found that TRHR-GFP DSGCs projected to the superficial-most sublamina of the SC and terminations were present along the full extent of the A-P axis of the SC in Isl2^+/+^; TRHR-GFP mice (Figure 1C). In contrast, terminals were found predominantly in the posterior SC of Isl2^EA3/EA3^; TRHR-GFP mice (Figure 1D). Interestingly, and consistent with previous data, the laminar targeting of TRHR-GFP RGC terminals was unaffected in the Isl2^EA3/EA3^ background (Triplett et al., 2014), suggesting endogenous layer-specific wiring mechanisms remain intact.

We next asked if more DSGCs in general projected to the posterior sub-region in Isl2^EA3/EA3^ mice. To do so, we retrogradely labeled RGCs projecting to the anterior and posterior domains by injecting fluorescently tagged cholera toxin subunit B (CTB-488) into specific sub-regions of the SC of adult Isl2^EA3/EA3^ mice (Figures 1E,E′,E″′,F,F′,F″′). Immunostaining for the On-Off DSGC marker CART (Kay et al., 2011) (Figures 1E″,E″′,F″,F″′) revealed that significantly more posterior-projecting RGCs were DSGCs compared to anterior-projecting RGCs in Isl2^EA3/EA3^ mice (Isl2^+/+^: 16.96 ± 1.66%, *n* = 3; Isl2^EA3/EA3^_ANT_: 11.63 ± 1.16%, *n* = 5; Isl2^EA3/EA3^_POST_: 23.19 ± 3.02%, *n* = 5; *P* = 0.0095, one-way ANOVA; *P* = 0.0074, Tukey’s *post hoc* analysis, Isl2^EA3/EA3^_ANT_ vs. Isl2^EA3/EA3^_POST_; Figure 1G). Together with our previous findings, these data suggest that the terminals of multiple DSGC subtypes are segregated preferentially into the posterior SC of Isl2^EA3/EA3^ mice.

### Asymmetric Distribution of DS Units in the SC of Isl2^EA3/EA3^ Mice

Based on the segregation of DSGC inputs into the posterior SC of Isl2^EA3/EA3^ mice and recent evidence demonstrating that direction-selectivity in the SC is inherited from retinal input (Shi et al., 2017), we predicted that DS units would be preferentially localized to the posterior SC in Isl2^EA3/EA3^ mice. To explore this possibility, we performed extracellular recordings from the superficial layers of the SC of Isl2^+/+^ and Isl2^EA3/EA3^ mice using high-density silicon multielectrodes while presenting visual stimuli. Of note, we utilized Isl2^+/+^ mice as controls instead of heterozygous Isl2^EA3/+^ mice, since we previously demonstrated that the organization of retinal inputs in the SC of Isl2^EA3/+^ mice is heterogeneous, both between animals and between hemispheres (Owens et al., 2015). We targeted recordings to the anterior or posterior region of the SC of both Isl2^+/+^ and Isl2^EA3/EA3^ mice and confirmed electrode location *post hoc*.

**Figure 2:**
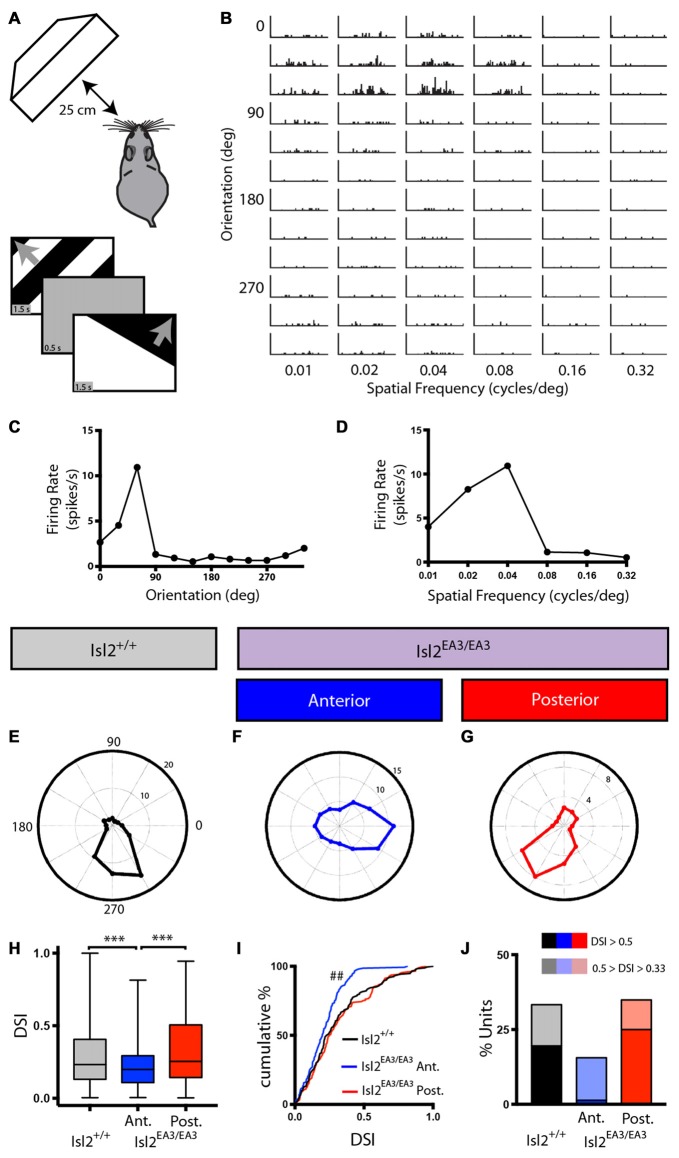
Asymmetric distribution of DS units in the SC of Isl2^EA3/EA3^ mice. **(A)** Schematic of visual stimulus setup. Anesthetized mice were presented drifting square wave stimulus moving in 12 different orientations and at six different spatial frequencies (SFs). **(B)** Representative peristimulus spike time histograms of spiking responses to square-wave gratings presented in the indicated orientations and SFs. **(C,D)** Quantification of firing rate as a function at each orientation **(C)** and SF **(D)** shown for the unit presented in **(B)** reveal tuning curves for each parameter. **(E–G)** Polar plots of representative DS units identified in the SC of Isl2^+/+^ mice **(E)** the anterior SC of Isl2^EA3/EA3^ mice **(F)** and the posterior SC of Isl2^EA3/EA3^ mice **(G)** where orientation is represented around the circumference of the plot and spike rate indicated by concentric rings. **(H)** Quantification of the average direction-selectivity index (DSI) of all units in each group. Boxes represent 25–75th percentile, line represents the median, and whiskers are minimum and maximum. ***p < 0.001, Kruskal-Wallis test with Dunn’s multiple comparisons test. **(I)** Quantification of the cumulative probability for all DSIs. ^##^*p* < 0.01, Kolmogorov-Smirnov test vs. Isl2^+/+^. **(J)** Quantification of the proportion of cells in each group classified as DS (orientation-selectivity index (OSI) ≥ 0.33, 0.5 ≥ DSI ≥ 0.33) and highly DS (OSI ≥ 0.33, DSI ≥ 0.5).

First, we presented drifting square waves of varying orientation and SFs (Figure 2A) and determined the spike rate in response to each possible combination (Figure 2B). Tuning curves were then generated to determine preferred orientations and SFs of identified units (Figures 2C,D). For each visually responsive unit, we calculated the OSI, which compares the response at the preferred orientation shown to the response at the orthogonal. We also calculated the DSI, which is a measure of the response at the preferred orientation to that at the opposite. Using these indices, we were able to classify neurons as AS (OSI ≥ 0.33 and DSI < 0.33) and DS (OSI ≥ 0.33 and DSI ≥ 0.33). We chose a threshold of 0.33 to define AS and DS units, which indicates a two-fold increase in spike rate at the preferred orientation to the comparison orientation and allows for comparison with previous studies (Wang et al., 2010).

We were able to identify DS units in each SC region we recorded from in both Isl2^+/+^ and Isl2^EA3/EA3^ mice, and representative polar plots of DS units are shown in Figures 2E–G. Strikingly, however, we found a significant decrease in the mean DSI for neurons in the anterior SC of Isl2^EA3/EA3^ mice (Isl2^+^ RGC-innervated, Isl2^EA3/EA3^_ANT_) compared to both Isl2^+/+^ and the posterior SC of Isl2^EA3/EA3^ mice (Isl2^−^ RGC-innervated, Isl2^EA3/EA3^_POST_; mean DSI = Isl2^+/+^: 0.3039 ± 0.01762, Isl2^EA3/EA3^_ANT_: 0.2107 ± 0.01119, Isl2^EA3/EA3^_POST_: 0.3191 ± 0.01751, *P* = 0.0003, Kruskal-Wallis test, Dunn’s multiple comparisons test: *P* = 0.0065, Isl2^EA3/EA3^_ANT_ vs. Isl2^+/+^; *P* = 0.0004, Isl2^EA3/EA3^_ANT_ vs. Isl2^EA3/EA3^_POST_; *P* > 0.9999, Isl2^EA3/EA3^_POST_ vs. Isl2^+/+^; Figure 2H). Units identified in the anterior and posterior SC of Isl2^+/+^ mice were grouped together, since we found no difference in any of the DS parameters measured, including DSI, tuning width, preferred SF, or linearity of response (data not shown).

In addition to a decreased mean DSI, we also found a significant difference in the cumulative probability curve for DSIs in Isl2^EA3/EA3^_ANT_ SC compared to both Isl2^+/+^ (*P* = 0.0018, Kolmogorov-Smirnov (K-S) test) and Isl2^EA3/EA3^_POST_ (*P* = 0.0002, K-S test; Figure 2I). Utilizing a DSI of ≥0.33 as the threshold for direction-selectivity, we determined the proportion of neurons that were classified as DS in each region and found a substantial reduction in the number of DS units in Isl2^EA3/EA3^_ANT_ SC (15.5%, 23/148) compared to Isl2^+/+^ (33.3%, 58/174) and Isl2^EA3/EA3^_POST_ (34.9%, 60/172; Figure 2J). Strikingly, only 1.4% (2/148) of neurons in Isl2^EA3/EA3^_ANT_ SC were classified as highly DS (DSI ≥ 0.5), which has previously been used as a cutoff for selectivity (Niell and Stryker, 2008), whereas 25% (43/172) of units in Isl2^EA3/EA3^_POST_ SC and 19.5% (34/174) in Isl2^+/+^ SC met this criteria. Taken together, these data suggest that neurons innervated solely by Isl2^−^ RGCs (Isl2^EA3/EA3^_POST_ SC) are more directionally tuned than those innervated solely by Isl2^+^ RGCs (Isl2^EA3/EA3^_ANT_ SC).

### Tuning Properties of DS Cells Innervated by Isl2^+^ and Isl2^−^ RGCs

**Figure 3:**
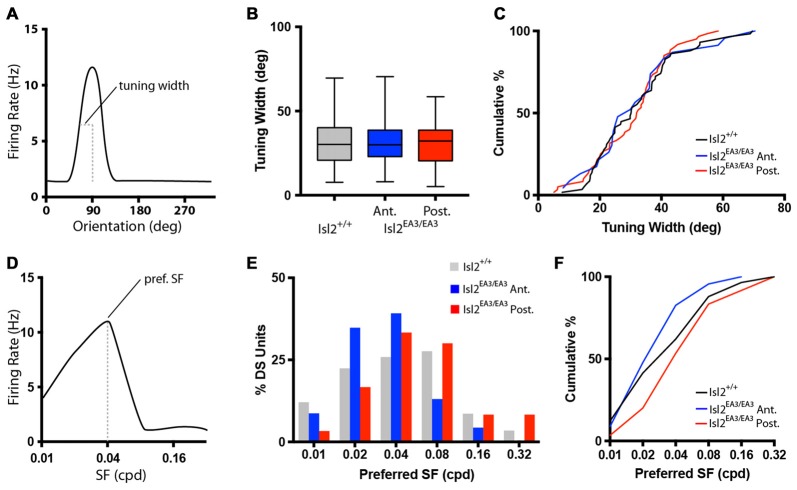
Similar tuning of DS units in the SC of Isl2^EA3/EA3^ mice. **(A)** Schematized orientation tuning curve of a DS unit indicating the tuning width parameter quantified in **(B,C)**. **(B)** Quantification of tuning width for DS units identified in each group. Boxes represent 25th–75th percentile, line represents the median, and whiskers are minimum and maximum. **(C)** Quantification of the cumulative probability for all tuning widths of DS units identified in each of the indicated groups. **(D)** Schematized SF tuning curve of a DS unit indicating the preferred SF parameter quantified in **(E,F)**. **(E)** Histogram of the percent of DS units preferring indicated SFs in each of the indicated groups. **(F)** Quantification of the cumulative probability for all preferred SFs of DS units identified in each of the indicated groups.

Given that the vast majority of DS neurons in the SC of Isl2^EA3/EA3^ mice were located in the posterior SC and innervated solely by Isl2^−^ RGCs, we next wondered if other properties of their tuning differed significantly from DS neurons found in the SC of Isl2^+/+^ mice. To begin, we analyzed the tuning width of DS neurons in each group, which reflects how sharply tuned to the preferred direction a given neuron is (Figure 3A). We found no difference in the average tuning width of neurons between groups (mean tuning width = Isl2^+/+^: 31.95 ± 1.820, Isl2^EA3/EA3^_ANT_: 31.91 ± 3.257, Isl2^EA3/EA3^_POST_: 30.67 ± 1.562, *P* = 0.9934, Kruskal-Wallis test; Figure 3B), nor did we observe a difference in the cumulative probability curves of tuning widths of DS units for each group (K-S test: *P* = 0.9750, Isl2^EA3/EA3^_ANT_ vs. Isl2^+/+^; *P* = 0.8760, Isl2^EA3/EA3^_ANT_ vs. Isl2^EA3/EA3^_POST_; *P* = 0.0.9235, Isl2^EA3/EA3^_POST_ vs. Isl2^+/+^; Figure 3C). These data demonstrate that DS neurons in Isl2^EA3/EA3^_ANT_ SC and Isl2^EA3/EA3^_POST_ SC exhibit similar sharpness of tuning to one another and to DS neurons in Isl2^+/+^ SC.

We next determined the preferred SF for each DS unit (Figure 3D), which is a reflection of the width of visual space to which a neuron is tuned. Consistent with previous studies (Wang et al., 2010), the preferred SF of DS neurons in the SC of Isl2^+/+^ mice ranged between 0.01 and 0.32 cycles/degree, with most preferring 0.02–0.08 (Figure 3E, *gray bars*). Interestingly, in Isl2^EA3/EA3^ mice, we found that this distribution was shifted slightly towards lower SFs for DS units in Isl2^EA3/EA3^_ANT_ SC (Figure 2E, *blue bars*), while the distribution of DS units in Isl2^EA3/EA3^_POST_ SC was similar to wild type (Figure 2E, *red bars*). This was reflected in the cumulative probability curve for preferred SFs for each group, with the Isl2^EA3/EA3^_ANT_ curve shifted slightly leftward (Figure 3F, *blue*); however, we did not observe a significant difference between these curves (K-S test: *P* = 0.4906, Isl2^EA3/EA3^_ANT_ vs. Isl2^+/+^; *P* = 0.1349, Isl2^EA3/EA3^_POST_ vs. Isl2^+/+^; *P* = 0.1157, Isl2^EA3/EA3^_ANT_ vs. Isl2^EA3/EA3^_POST_; Figure 3F). These data suggest that the DS units innervated by Isl2^+^ and Isl2^−^ exhibit similar SF tuning to those found in Isl2^+/+^ SC.

**Figure 4:**
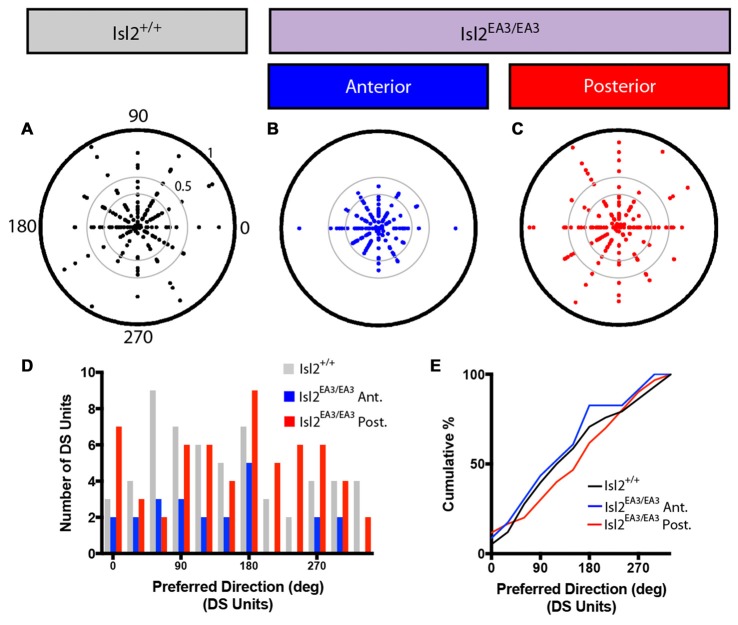
Distribution of preferred directions for DS units is unchanged in the SC of Isl2^EA3/EA3^ mice. **(A–C)** Polar plots of preferred angle and corresponding DSI for all units identified in the SC of Isl2^+/+^ mice **(A)** the anterior **(B)** and posterior **(C)** SC of Isl2^EA3/EA3^ mice. Orientation is indicated around the circumference and DSI increases from 0 to 1 centrifugally. DSIs of 0.33 and 0.5 are indicted by concentric circles. **(D)** Histogram of the number of DS units preferring indicated directions in each of the indicated groups. **(E)** Quantification of the cumulative probability for all preferred directions of DS units identified in each of the indicated groups.

We next asked if DS neurons in each group might be preferentially tuned to particular directions. To do so, we first plotted the preferred direction of each neuron around a polar plot, with DSI increasing centrifugally (Figures 4A–C). These plots reveal that DS neurons preferring any of the 12 directions presented could be found in each group, and there appeared to be no relation between preferred direction and DSI. To demonstrate this in another way, we examined the distribution of preferred directions and found that there was no bias for any particular preferred direction(s) for DS units in Isl2^+/+^ nor Isl2^EA3/EA3^ SC (Figure 4D). Consistent with this, there was no difference in the cumulative probability curves for preferred direction between any of the groups (K-S test: *P* = 0.9734, Isl2^EA3/EA3^_ANT_ vs. Isl2^+/+^; *P* = 0.7933, Isl2^EA3/EA3^_POST_ vs. Isl2^+/+^; *P* = 0.4594, Isl2^EA3/EA3^_ANT_ vs. Isl2^EA3/EA3^_POST_; Figure 4E). These data suggest that DS neurons in both domains of the SC in Isl2^EA3/EA3^ mice and in Isl2^+/+^ mice prefer a broad range of directions.

**Figure 5:**
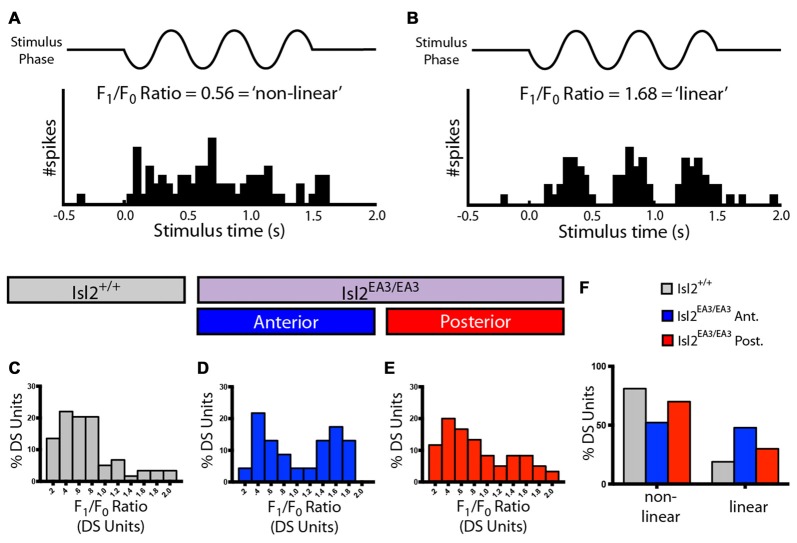
Spatial summation properties of DS units in the SC of Isl2^EA3/EA3^ mice. **(A,B)** Peristimulus spike time histograms of representative linear **(A)** and non-linear **(B)** DS units in response to drifting square wave stimulus presented in the preferred orientation. Ratio of the response at the stimulus frequency (F_1_), indicated by to the mean response (F_0_), a measure of spatial summation properties of the neuron, is indicated for each representative neuron. **(C–E)** Histograms of the relative distribution of F_1_/F_0_ ratio in bins of 0.2 for DS units identified in the SC of Isl2^+/+^ mice **(C)** the anterior **(D)** and posterior **(E)** SC of Isl2^EA3/EA3^ mice. **(F)** Quantification of the proportion of DS units classified as non-linear (F_1_/F_0_ < 1) and linear (F_1_/F_0_ > 1) in each of the indicated groups.

Finally, we determined the spatial summation properties of DS neurons in the SC for each group by measuring the F_1_/F_0_ ratio, which relates the firing rate at the temporal frequency of the stimulus to the mean rate (Figures 5A,B). Traditionally, an F_1_/F_0_ value greater than 1 is commonly considered a linear response, and less than 1 is non-linear (Skottun et al., 1991). Consistent with previous data (Wang et al., 2010), we found that most DS neurons in the SC of Isl2^+/+^ mice were non-linear, having an F_1_/F_0_ ratio < 1 (73.00%, 47/58; Figures 5C,F). Interestingly, we found that the distribution of F_1_/F_0_ ratios for DS neurons in Isl2^EA3/EA3^_ANT_ SC was bimodal, and substantially more were linear compared to Isl2^+/+^ or Isl2^EA3/EA3^_POST_ SC (Isl2^+/+^ 18.97%, 11/58; Isl2^EA3/EA3^_ANT_ 47.83%, 11/23; Isl2^EA3/EA3^_POST_ 30.00%, 18/60; Figures 5C–F). These data suggest that DS neurons innervated by Isl2^+^ RGCs may be more linear compared to DS neurons in Isl2^EA3/EA3^_POST_ or Isl2^+/+^ SC. However, we found no difference in the median F_1_/F_0_ ratio between Isl2^EA3/EA3^_ANT_ SC and Isl2^+/+^ SC or Isl2^EA3/EA3^_POST_ (*P* = 0.3306, Kruskal-Wallis test). Overall, our analysis suggests that despite being in lower abundance and less selective, DS neurons innervated by Isl2^+^ RGCs have similar SF tuning and spatial summation properties compared to those innervated by Isl2^−^ RGCs and those in Isl2^+/+^ SC.

### Asymmetric Distribution of Tightly Tuned AS Units in the SC of Isl2^EA3/EA3^ Mice

**Figure 6:**
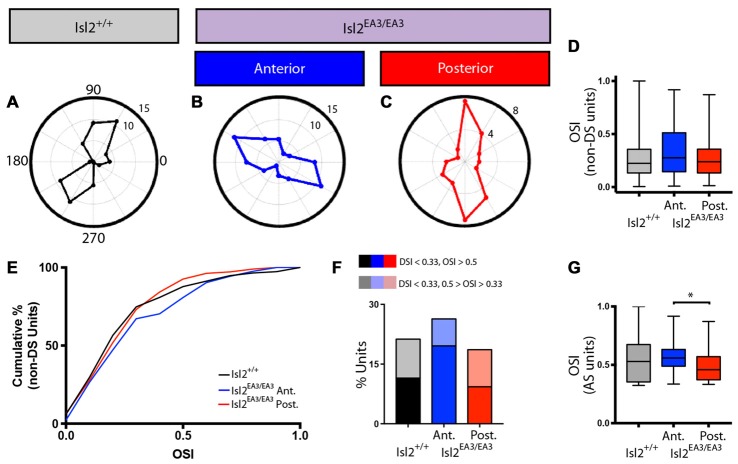
Increased proportion of tightly tuned AS units in the anterior SC of Isl2^EA3/EA3^ mice.** (A–C)** Representative polar plots of AS units identified in the SC of Isl2^+/+^ mice **(A)** the anterior **(B)** and posterior **(C)** SC of Isl2^EA3/EA3^ mice. Orientation of stimulus is indicated around the plot and spike rates are indicated on concentric circles. **(D)** Quantification of the average OSI of non-DS units in each group. Boxes represent 25–75th percentile, line represents the median, and whiskers are minimum and maximum. **(E)** Quantification of the cumulative probability for OSIs of non-DS units in each of the indicated groups. **(F)** Quantification of the proportion of cells in each group classified as AS (0.5 ≥ OSI ≥ 0.33, DSI < 0.33) and highly AS (OSI ≥ 0.5, DSI < 0.33). **(G)** Quantification of the average OSI of non-DS units in each group. Boxes represent 25–75th percentile, line represents the median, and whiskers are minimum and maximum. *p < 0.05, Kolmogorov-Smirnov test.

In addition to identifying DS units with our drifting square-wave stimulus, we were also able to identify AS units in each of the groups analyzed, and representative polar plots are presented in Figures 6A–C. Given the differences in DSI observed between Isl2^EA3/EA3^_ANT_ SC and Isl2^EA3/EA3^_POST_ SC, we wondered if differences in OSI might also be found. In order to examine specifically the prevalence of AS units, we removed DS units from this analysis, since by our definition they have an OSI ≥ 0.33 and substantially more are found in Isl2^EA3/EA3^_POST_ SC. Despite this, we did not observe a difference between any of the groups (mean OSI (non-DS units) = Isl2^+/+^: 0.287 ± 0.0207, Isl2^EA3/EA3^_ANT_: 0.3245 ± 0.0200, Isl2^EA3/EA3^_POST_: 0.2735 ± 0.0178, *P* = 0.2811, Kruskal-Wallis test; Figure 3B), although the distribution of OSIs appeared to be shifted slightly higher for AS units in Isl2^EA3/EA3^_ANT_ SC (Figure 6D, *blue*). Consistent with this, we observed a slightly rightward shift in the cumulative probability curve for OSIs of AS units in Isl2^EA3/EA3^_ANT_ SC (Figure 6E); however, we did not observe a significant difference between any groups (K-S test: *P* = 0.3078, Isl2^EA3/EA3^_ANT_ vs. Isl2^+/+^; *P* = 0.8235, Isl2^EA3/EA3^_POST_ vs. Isl2^+/+^; *P* = 0.1093, Isl2^EA3/EA3^_ANT_ vs. Isl2^EA3/EA3^_POST_). Units identified in the anterior and posterior SC of Isl2^+/+^ mice were grouped together, since we found no difference in any of the AS parameters measured, including OSI, tuning width, preferred SF, or linearity of response (data not shown).

Utilizing an OSI of 0.33 as the threshold for axis-selectivity, we observed a slight increase in the proportion of units that were AS in Isl2^EA3/EA3^_ANT_ SC (26.35%, 39/148) compared to Isl2^EA3/EA3^_POST_ SC (18.6%, 32/172) and Isl2^+/+^ SC (21.26%, 37/174; Figure 6F). This increase was particularly apparent for highly selective AS units (OSI ≥ 0.5, DSI < 0.33), as we found that 19.6% (29/148) units were highly AS in Isl2^EA3/EA3^_ANT_ SC compared to 9.3% (16/172) in Isl2^EA3/EA3^_POST_ SC and 11.5% (20/174) in Isl2^+/+^ SC (Figure 6F). Consistent with this, we found a significant difference in the OSI of AS units in Isl2^EA3/EA3^_ANT_ SC compared to Isl2^EA3/EA3^_POST_ SC (K-S test: *P* = 0.1573, Isl2^EA3/EA3^_ANT_ vs. Isl2^+/+^; *P* = 0.5745, Isl2^EA3/EA3^_POST_ vs. Isl2^+/+^; *P* = 0.035, Isl2^EA3/EA3^_ANT_ vs. Isl2^EA3/EA3^_POST_; Figure 6G). Taken together, these data suggest AS neurons innervated by Isl2^+^ RGCs tend to be more axially tuned than those innervated by Isl2^−^ RGCs in Isl2^EA3/EA3^ SC.

### Tuning of AS Cells Innervated by Isl2^+^ and Isl2^−^ RGCs in Isl2^EA3/EA3^ Mice

**Figure 7:**
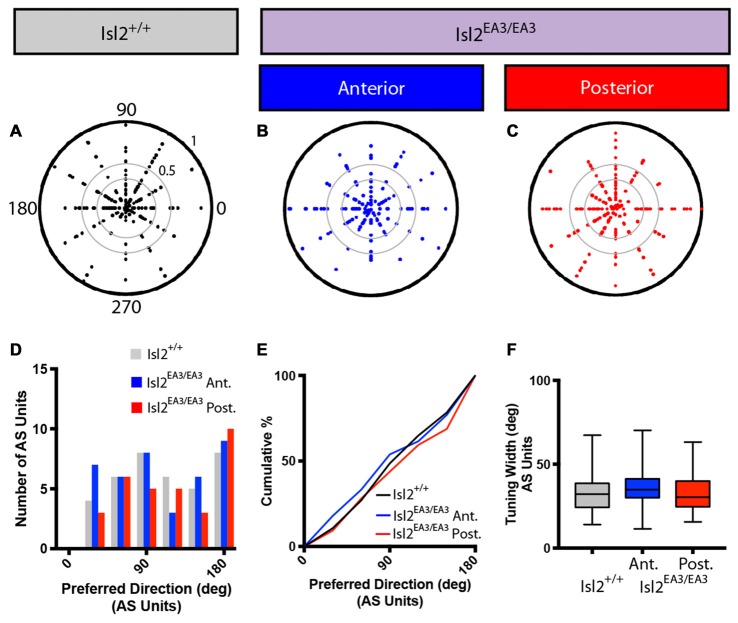
Distribution of preferred orientations and tuning width for AS units are unchanged in the SC of Isl2^EA3/EA3^ mice.** (A–C)** Polar plots of preferred angle and corresponding OSI for all non-DS units identified in the SC of Isl2^+/+^ mice **(A)** the anterior **(B)** and posterior **(C)** SC of Isl2^EA3/EA3^ mice. Preferred orientation is indicated around the circumference and OSI increases from 0 to 1 centrifugally. OSIs of 0.33 and 0.5 are indicted by concentric circles. **(D)** Histogram of the number of AS units preferring indicated orientations in each of the indicated groups. **(E)** Quantification of the cumulative probability for all preferred orientations of AS units identified in each of the indicated groups. **(F)** Quantification of tuning width for AS units identified in each group. Data are presented as average ± SEM and individual values indicated as dots.

We next wondered if the tuning properties of AS neurons in each sub-region were different. We first determined the preferred orientation of AS neurons in each group and found no bias for preference to any particular orientation between any of the groups, as evidenced qualitatively by examining polar plots of preferred orientation as a function of OSI, the distribution of preferred orientations, and the cumulative probability of preferring any orientation (K-S test: *P* > 0.9999, Isl2^EA3/EA3^_ANT_ vs. Isl2^+/+^; *P* = 0.9973, Isl2^EA3/EA3^_POST_ vs. Isl2^+/+^; *P* = 0.9939, Isl2^EA3/EA3^_ANT_ vs. Isl2^EA3/EA3^_POST_; Figures 7A–E). We next determined the tuning width of AS neurons, and, similar to DS cells, we found no difference between AS neurons innervated by Isl2^+^ or Isl2^−^ RGCs (mean tuning width = Isl2^+/+^: 34.14 ± 2.194, Isl2^EA3/EA3^_ANT_: 36.94 ± 1.994, Isl2^EA3/EA3^_POST_: 32.24 ± 2.084, *P* = 0.2872, one-way ANOVA; Figure 7F). Importantly, the tuning width of AS neurons in each subdomain was similar to that observed for AS neurons in the SC of Isl2^+/+^ mice, suggesting that similar types of RFs are constructed despite the dramatic rewiring of RGC inputs to the SC in Isl2^EA3/EA3^ mice.

**Figure 8:**
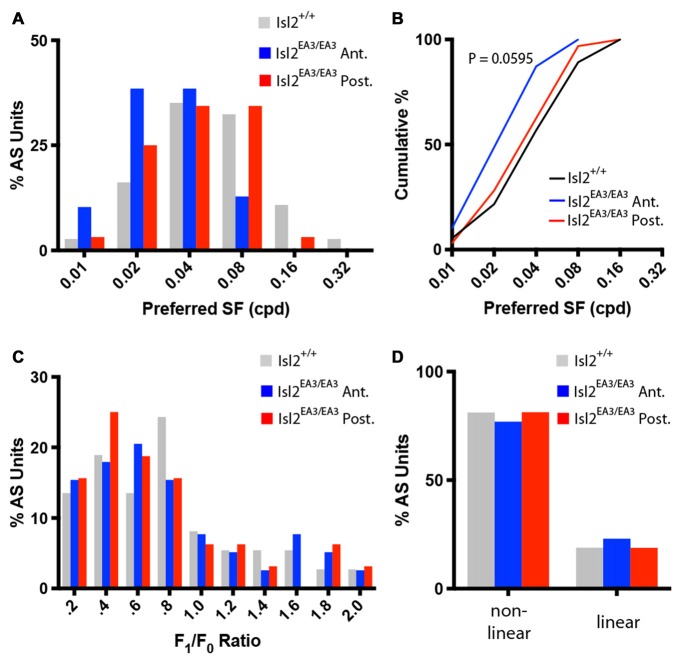
Similar SF tuning and spatial summation properties of AS units in the anterior and posterior SC of Isl2^EA3/EA3^ mice.** (A)** Histogram of the percent of AS units preferring indicated SFs in each of the indicated groups. **(B)** Quantification of the cumulative probability for all preferred SFs of AS units identified in each of the indicated groups. **(C)** Histograms of the percent of AS units with F_1_/F_0_ ratio within indicated bins of 0.2 identified in the SC of the indicated groups. **(D)** Quantification of the proportion of DS units classified as non-linear (F_1_/F_0_ < 1) and linear (F_1_/F_0_ > 1) in each of the indicated groups.

Next, we determined the preferred SF of AS neurons. Consistent with previous data, we found that most AS neurons in the SC of Isl2^+/+^ mice prefer SFs between 0.04 and 0.08 cycles/degree (Wang et al., 2010; Figure 8A, *gray bars*). Interestingly, we found that this distribution was shifted towards lower SFs for AS neurons in Isl2^EA3/EA3^_ANT_ SC (Figure 8A, *blue bars*), but not for AS neurons in Isl2^EA3/EA3^_POST_ SC (Figure 8A, *red bars*). This was reflected in a shift of the cumulative probability curve for preferred SF of AS neurons in Isl2^EA3/EA3^_ANT_ SC compared to Isl2^+/+^ or Isl2^EA3/EA3^_POST_ SC (Figure 8B); however, this was not a statistically significant difference, though it trended towards significance (K-S test: *P* = 0.0595, Isl2^EA3/EA3^_ANT_ vs. Isl2^+/+^; *P* > 0.9999, Isl2^EA3/EA3^_POST_ vs. Isl2^+/+^; *P* = 0.2346, Isl2^EA3/EA3^_ANT_ vs. Isl2^EA3/EA3^_POST_).

Finally, we determined the spatial summation properties of AS neurons in each group. Similar to DS neurons and previous reports (Wang et al., 2010), the vast majority of AS neurons in the SC of Isl2^+/+^ mice were non-linear, having an F_1_/F_0_ ratio less than 1 (Figures 8C,D, *gray bars*). We found that the F_1_/F_0_ ratios of AS neurons in Isl2^EA3/EA3^_ANT_ SC were not bimodally distributed, as most were non-linear (Figures 8C,D, *blue bars*). We found the same to be true for AS neurons in Isl2^EA3/EA3^_POST_ SC (Figures 8C,D, *red bars*). These data suggest that spatial summation properties of AS neurons innervated by Isl2^+^ and Isl2^−^ RGCs in Isl2^EA3/EA3^ mice are similar and resemble those of AS units identified in the SC of Isl2^+/+^ mice. Overall, our analysis suggests that AS neurons innervated by Isl2^+^ RGCs in the SC of Isl2^EA3/EA3^ mice are more selective than those innervated by Isl2^−^ RGCs or AS units found in Isl2^+/+^ SC.

### Receptive Field Size and Structure Are Similar for Neurons Innervated by Islet2^+^ and Islet^−^ RGCs

**Figure 9:**
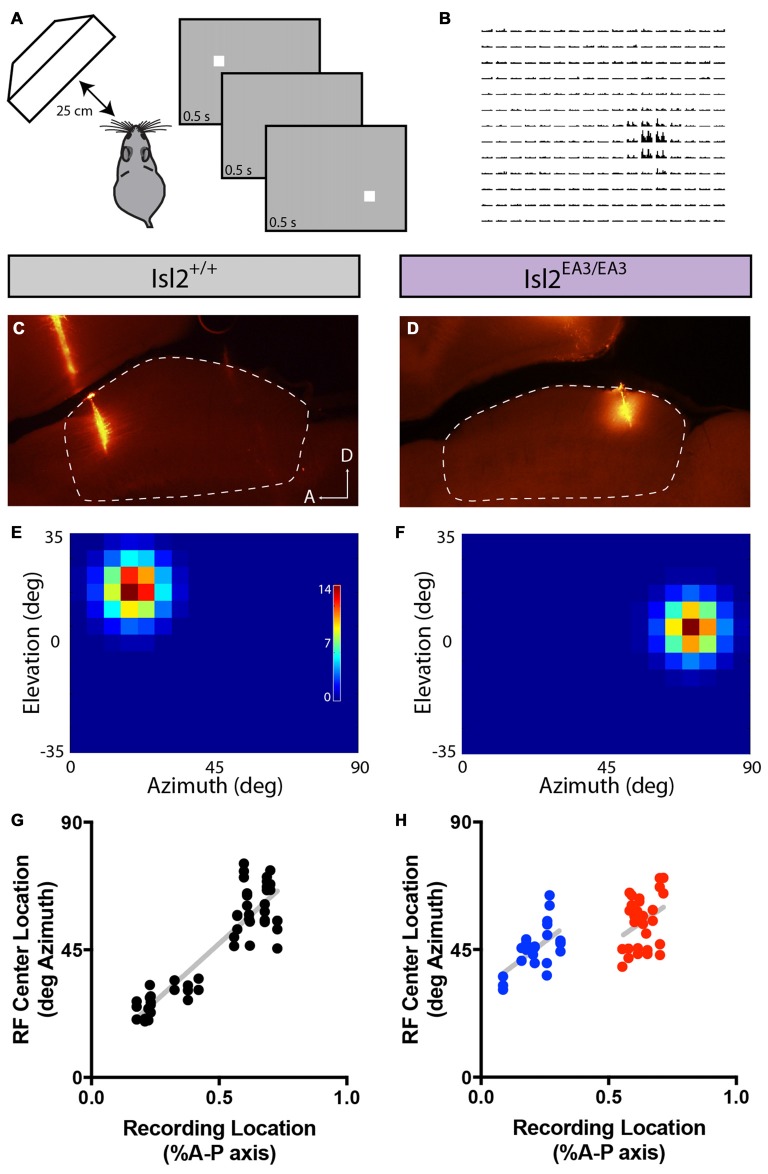
Altered topography of visual neurons in the SC of Isl2^EA3/EA3^ mice.** (A)** Schematic of visual stimulus and recording paradigm. Anesthetized mice were presented a flashing spot of 5 degree^2^. **(B)** Peristimulus spike time histograms for each potential position of the spot illustrate a representative response to the flashing spot stimulus. **(C,D)** Fluorescent images of parasagittal sections through the SC of Isl2^+/+^ **(C)** or Isl2^EA3/EA3^ **(D)** reveal the track and recording location of electrodes inserted in the SC (dashed area). *A, anterior; D, dorsal*. **(E,F)** Response heat maps to flashing spot stimulus of units identified from recording locations in **(C,D)**. **(G,H)** Plots of RF center location along the azimuth axis of visual space as a function of recording location along the A-P axis of the SC.

We next wondered what the RF size or structure of SC neurons innervated by Isl2^+^ and Isl2^−^ RGCs was. To determine RF size and structure, we presented a flashing white spot of 5° × 5° on a gray background on a screen subtending ~90° × 70° of visual space (Figure 9A) and determined the number of spikes elicited when the spot was presented in each location (Figure 9B). In both Isl2^+/+^ and Isl2^EA3/EA3^ mice, we were able to identify visually responsive neurons and reconstruct their RFs in each recording location (Figures 9C–F). We first asked if the alterations in retinotopic order of visual responses in the SC of Isl2^EA3/EA3^ mice were consistent with our previous intrinsic signal optical imaging studies (Triplett et al., 2009). To do so, we plotted the location of the RF center along the azimuth axis of space as a function of recording location along the A-P axis of the SC. In Isl2^+/+^ mice, neurons monitoring central visual space were found anteriorly, with a smooth progression to those monitoring peripheral visual space as recordings were performed more posteriorly (Figure 9G). In contrast, in Isl2^EA3/EA3^ mice, the progression along the azimuth extent is represented in both the anterior and posterior halves of the SC (Figure 9H). These data are consistent with previous intrinsic signal optical imaging studies (Triplett et al., 2009), and confirm that the topographic map of azimuth in the SC of Isl2^EA3/EA3^ mice is duplicated along the A-P axis.

**Figure 10:**
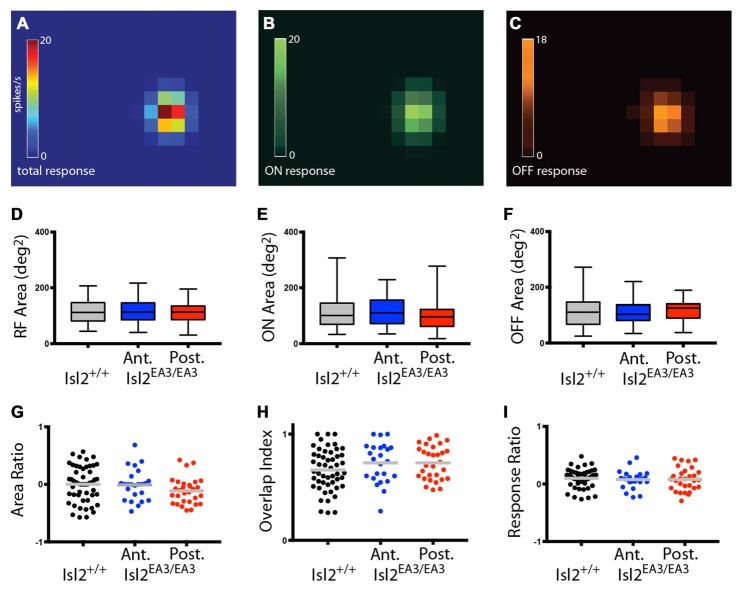
Visual neurons in the anterior and posterior SC of Isl2^EA3/EA3^ mice exhibit similar response properties to flashing spot stimulus. **(A–C)** Heat maps of the total response **(A)** ON response **(B)** and OFF response **(C)** to spot stimulus of a representative SC neuron. **(D–F)** Quantification of the total RF area **(D)** ON area **(E)** and OFF area **(F)**. Boxes represent 25–75th percentile, line represents the median, and whiskers are minimum and maximum. **(G–I)** Quantification of the ON/OFF area ratio **(G)** overlap index **(H)** and peak ON/peak OFF response ratio **(I)**. Each point is an individual unit and the bars represent mean.

We next wanted to determine if there were differences in the size of RFs innervated by Isl2^+^ and Isl2^−^ RGCs. Previous studies demonstrated that most neurons in the SC display both On and Off responses to stimuli presented anywhere in their RF (Drager and Hubel, 1975; Wang et al., 2010), though the optimal stimulus size and response properties vary between subtypes of SC neuron (Gale and Murphy, 2014). While we did not determine optimal stimulus size, we did find that the vast majority of neurons in the SC of both Isl2^+/+^ and Isl2^EA3/EA3^ mice were responsive to both On and Off stimuli throughout their RF (Figures 10A–C). We calculated the area of the total RF, as well as the On and Off subdomains and found no difference between units innervated exclusively by Isl2^+^ or Isl2^−^ RGCs in the SC of Isl2^EA3/EA3^ mice compared to one another or to those found in Isl2^+/+^ controls (mean total RF area (degree^2^) = Isl2^+/+^: 115.9 ± 6.377, Isl2^EA3/EA3^_ANT_: 113.7 ± 8.755, Isl2^EA3/EA3^_POST_: 107.4 ± 7.02, *P* = 0.6900, one-way ANOVA; mean On RF area = Isl2^+/+^: 115.5 ± 8.62, Isl2^EA3/EA3^_ANT_: 114.1 ± 11.42, Isl2^EA3/EA3^_POST_: 98.5 ± 9.48, *P* = 0.5331, Kruskal-Wallis test; mean Off RF area = Isl2^+/+^: 116.3 ± 8.355, Isl2^EA3/EA3^_ANT_: 113.3 ± 10.73, Isl2^EA3/EA3^_POST_: 116.4 ± 7.421, *P* = 0.9708, one-way ANOVA; Figures 10D–F). To determine the relative size of On and Off subfields, we calculated the Area Ratio, and again found no difference between groups (mean Area Ratio = Isl2^+/+^: 0.002215 ± 0.04334, Isl2^EA3/EA3^_ANT_: −0.007291 ± 0.05556, Isl2^EA3/EA3^_POST_: −0.1135 ± 0.04151, *P* = 0.1796, one-way ANOVA; Figure 10G). These data demonstrate that RF sizes of visual neurons in the SC of Isl2^EA3/EA3^ mice are unchanged despite the dramatic reorganization of retinal inputs and suggest that changes in SF tuning are not likely due to alterations in RF size.

Finally, we asked if there were changes in the structure of RFs in the SC of Isl2^EA3/EA3^ mice. To do so, we determined the overlap index, which is a measure of the degree to which On and Off subfields are congruent, where an index of 1 indicates complete overlap and 0 indicates no overlap. Similar to previous reports, we found that most neurons in the SC of Isl2^+/+^ mice had a high overlap index, and we found no difference in either Isl2^EA3/EA3^_ANT_ SC or Isl2^EA3/EA3^_POST_ SC (mean overlap index = Isl2^+/+^: 0.6622 ± 0.02723, Isl2^EA3/EA3^_ANT_: 0.7286 ± 0.03844, Isl2^EA3/EA3^_POST_: 0.7292 ± 0.02872, *P* = 0.1803, one-way ANOVA; Figure 10H). We next determined the relative strength of the On and Off responses by calculating the Response Ratio. We found that the response to both On and Off stimuli was relatively similar (Response Ratio = ~0) for units in the SC of Isl2^+/+^ mice, and we observed no difference in either Isl2^EA3/EA3^_ANT_ SC or Isl2^EA3/EA3^_POST_ SC (mean Response Ratio = Isl2^+/+^: 0.09856 ± 0.02222, Isl2^EA3/EA3^_ANT_: 0.07597 ± 0.03226, Isl2^EA3/EA3^_POST_: 0.08223 ± 0.03603, *P* = 0.8406, one-way ANOVA; Figure 10I). Importantly, we also found no difference in any of these parameters when comparing units from the anterior and posterior SC of control Isl2^+/+^ mice (data not shown). Taken together, these data suggest that despite the dramatic rearrangement of RGC inputs in the SC of Isl2^EA3/EA3^ mice, neurons innervated exclusively by Isl2^+^ or Isl2^−^ RGCs exhibit similar responses to a flashing spot stimulus as those found in the SC of Isl2^+/+^ mice and that differences in preferred SF and linearity are not a result of altered RF size or structure.

## Discussion

Deciphering the pattern of inputs that give rise to a neuron’s tuning properties is critical for understanding the complex neural computations executed in the brain. In this study, we have investigated how molecularly distinct RGCs may contribute to the tuning properties of visual neurons in the SC. To do so, we have leveraged a unique mouse model in which the projections of Isl2^+^ and Isl2^−^ RGCs have been segregated into the anterior and posterior sub-regions of the SC, respectively, and found asymmetric distributions of DS units between these regions. These findings demonstrate that innervation by Isl2^−^ RGCs may be preferentially utilized to drive direction-selectivity in the SC. Interestingly, we found that either Isl2^+^ or Isl2^−^ RGCs can drive axis-selectivity in the SC; however, similar to DS neurons, the selectivity of AS neurons innervated by Isl2^+^ RGCs was increased compared to those innervated by Isl2^−^ RGCs. Despite the dramatic rearrangement of RGC inputs, tuning properties of DS and AS neurons, such as SF preference and spatial summation were similar in neurons innervated by Isl2^+^ and Isl2^−^ RGCs. Further, the size and structure of RFs were also similar. Taken together, these findings inform our understanding of how distinct RF properties may be established in the SC and lay the foundation for future studies elucidating the molecular mechanisms by which the synaptic specificity required for such exquisite tuning is established.

### Direction-Selectivity in the SC May Be Preferentially Built from Isl2^−^ RGC Inputs

Recent studies suggest that dedicated networks of direction-selectivity exist throughout the visual system, including a dedicated retina-dLGN-V1 circuit and a SC-dLGN pathway (Cruz-Martín et al., 2014; Bickford et al., 2015). The existence of such networks underscores the importance of establishing direction-selectivity, however the molecular logic underlying such connectivity is unclear. Recent work has demonstrated that direction-selectivity in the SC is inherited from the retina (Shi et al., 2017), but whether DSGC inputs are sufficient to drive direction-selectivity remains an open question. Here, we find that highly DS neurons are primarily encountered in the posterior domain of the SC in Isl2^EA3/EA3^ mice, implicating that Isl2^−^ RGC inputs play a critical role in establishing direction-selectivity. Consistent with this, we previously showed that a subset of Isl2^−^ RGCs have dendritic branching morphology consistent with that of On-Off DSGCs (Triplett et al., 2014). Further, Isl2^−^ RGC-innervated DS neurons exhibited non-linear spatial summation, suggesting a direct relay of On-Off response properties in the SC. Importantly, while these data suggest direction-selectivity in the SC is relayed from the retina, it is not likely to be via a strict “labeled-line” mechanism, since DS neurons in both Isl2^+^ and Isl2^−^ RGC-innervated regions exhibit a wider range of preferred directions in comparison to On or On-Off DSGCs, which are selective for movement along the cardinal axes (Vaney et al., 2012; Dhande et al., 2013; Fiscella et al., 2015; Bos et al., 2016). Thus, combinations of different cardinally tuned DSGCs are likely integrated to establish new preferred directions, but how these intermediate preferences are achieved remains unclear.

One potential limitation of our interpretation of these data is the effect of overexpression of EphA3 in the retina of Isl2^EA3/EA3^ mice on the tuning properties of both Isl2^+^ and Isl2^−^ RGCs. Intriguingly, we did not observe any changes in the proportion, dendritic lamination patterns, or projection patterns (other than topographic order in the SC) of genetically-labeled RGCs in this or previous studies (Triplett et al., 2014), suggesting that functionality may be maintained for RGCs in these transgenic mice. Consistent with this, similar properties of genetically-labeled RGCs are unaffected in other manipulations of EphA/ephrin-A signaling, such as loss-of-function via ephrin-A2/A3/A5 knockout (Sweeney et al., 2015). However, we cannot exclude the possibility that the alterations in tuning properties observed in the different regions of the SC in Isl2^EA3/EA3^ mice may be an artifact of the transgenic manipulation. Future studies employing retrograde tracing strategies and functional analysis of labeled RGCs projecting to each region are needed to address this issue.

### Axis-Selectivity May Be Constructed from Isl2^+^ or Isl2^−^ RGC Inputs in the SC

In the SC, orientation tuning most resembles that of complex orientation-selective (OS) cells in primary visual cortex (V1; Niell and Stryker, 2008) and AS cells in the dLGN (Marshel et al., 2012), since they have overlapped On- and Off-subdomains and exhibit non-linear spatial summation (Wang et al., 2010). Our data suggest that such tuning can arise via innervation from either Isl2^+^ or Isl2^−^ inputs, but what might be the exact wiring diagram? We previously showed that Isl2^+^ RGCs are comprised of multiple subtypes, which share the common morphological feature of mono-stratification in the IPL of the retina (Triplett et al., 2014). Additionally, a high proportion of RGCs express the αRGC marker SMI-32, which have On- or Off-center-surround RFs (Wässle et al., 1983). Thus, it is possible that a subset of AS neurons in the SC derive their tuning via a model similar to that proposed by Hubel and Wiesel (Hubel and Wiesel, 1962). However, the non-linearity of AS neurons in the Isl2^+^ RGC-innervated region of the SC in Isl2^EA3/EA3^ mice suggests that additional processes shape tuning. One possible circuit may involve local inhibitory connectivity from surrounding AS cells that prefer different axes, as has been proposed for some complex OS cells in V1 (Sillito, 1979). In support of this, recent work suggests that different regions of the mouse SC may be tuned to distinct orientations (Ahmadlou and Heimel, 2015; Feinberg and Meister, 2015), raising the possibility that lateral connections may shape these domains. Further, a role for local inhibitory connections has recently been uncovered for the detection of object motion (Gale and Murphy, 2016). However, future investigations of the influence of inhibitory circuitry on orientation-selectivity in the SC are needed to test this model.

Alternatively, axis-selectivity in the SC may be inherited from the retina, similar to the current model for direction-selectivity (Shi et al., 2017). Indeed, recent studies have identified OS RGCs in the mouse retina (Chen et al., 2014; Baden et al., 2016; Nath and Schwartz, 2016), and labeled line mechanisms have recently been shown to be capable of underlying the transfer of orientation-selectivity between the dLGN and V1 in the mouse (Sun et al., 2015). Indeed, estimates of the proportion of RGCs that are OS range between 14.5% and 19.5% (Chen et al., 2014; Baden et al., 2016), while we and others find between 22.25% and ~30% in the SC (Wang et al., 2010). However, genetic markers for OS RGCs have yet to be developed, so the projection patterns and molecular identity of these cells are unknown.

We also observed AS neurons in the posterior SC of Isl2^EA3/EA3^ mice, which are innervated by Isl2^−^ RGCs. As discussed above, a substantial portion of Isl2^−^ RGCs resemble On-Off DSGCs morphologically, raising the possibility that AS cells in the SC may also arise via convergent innervation by oppositely tuned DSGCs. Interestingly, a high prevalence of OS and AS neurons are observed in a region of the dLGN densely innervated by DSGCs (Marshel et al., 2012; Piscopo et al., 2013), suggesting that selectivity may arise via the convergent innervation of DSGCs tuned to opposing directions of movement. However, not all Isl2^−^ RGCs are DSGCs, and more work leveraging novel genetic tools that can label subpopulations of neurons in the SC are needed to clarify which mechanism is used to establish axis-selectivity.

## Conclusion

In this study, we show that innervation by molecularly distinct RGCs results in distinct tuning properties in the SC. Specifically, Isl2^−^ RGCs are utilized to construct DS neurons, while both Isl2^+^ and Isl2^−^ RGCs are utilized to construct AS RFs, though the selectivity and spatial tuning of each differ. In addition to beginning to elucidate the functional retinocollicular wiring diagram, these experiments also lay the groundwork for future investigations of the mechanisms by which specific synaptic connections are established in the SC.

## Author Contributions

RBK aided in the design of experiments; acquired, analyzed and/or interpreted all of the data; aided in the writing and revision of the manuscript. JWT conceived the study and designed the experiments; acquired, analyzed, and/or interpreted all of the data; wrote and edited the manuscript.

## Conflict of Interest Statement

The authors declare that the research was conducted in the absence of any commercial or financial relationships that could be construed as a potential conflict of interest.
